# Diagnosis and Management of Pregnant Women With Placental Abruption and Neonatal Outcomes

**DOI:** 10.7759/cureus.21120

**Published:** 2022-01-11

**Authors:** Souhail Alouini, Antoine Valery, Bruno Lemaire, Marie-Liesse Evrard, Olivier Belin

**Affiliations:** 1 Obstetrics and Gynecologic Surgery, Centre Hospitalier Regional d'Orleans, Orleans, FRA; 2 Bioinformatics, Centre Hospitalier Regional d'Orleans, Orleans, FRA; 3 Obstetrics and Gynaecology, Center Hospitalier Regional d'Orleans, Orleans, FRA; 4 Anesthesiology and Critical Care, Center Hospitalier Regional d'Orleans, Orleans, FRA

**Keywords:** foetal death, caseraen delivery, delivery time, foetal acidosis, umbilical ph, placenta abruptio

## Abstract

Background

Placenta abruptio (PA) remains a serious materno-fetal complication. According to progress realized in maternal-fetal medicine, we aimed to evaluate the diagnosis and management of PA and neonatal outcomes.

Methods

We conducted a retrospective study that involved all the patients that were diagnosed with PA in a tertiary maternity hospital between 2006 and 2013. Data were analyzed to determine mean and standard deviation and statistically analyzed using the Chi-square test.

Results

In total, 201 patients were diagnosed with PA out of 35184 deliveries (0.56%). The mean age of patients was 30 years and most of them were multiparous (56.2%). Thirty-six out of 201 patients (17.9%) smoked tobacco or consumed alcohol during the pregnancy. Three patients came from their homes. Twenty-eight patients had preeclampsia and 105 presented with high blood pressure. Furthermore, 117 patients presented metrorrhagia (58.2%) and 39% of patients exhibited abdominal pain. We reported fetal heart rate abnormalities in 57% of the cases. Ultrasound examination revealed PA in only 48 patients (23.9%). One hundred eighty out of 201 patients (84.6%) underwent an emergency caesarean section. One hundred sixty-seven fetuses were born prematurely. Thirteen out of 201 fetuses died, and 98 newborns needed neonatal resuscitation. In total, 31 fetuses had an umbilical artery (UA) with pH ≤ 7 (31/188). The mean time for delivery was 18.7 min. However, UA pH did not differ when the delivery time was shorter (p = 0.09). Seventy-six percent of cases came from their homes. The mean UA pH was significantly lower for PA cases who came from their homes compared to hospitalized women (p = 0.0015). Histological examination of the placenta confirmed the diagnosis in 71 out of 148 cases (47.9%). The mean duration of hospital stay of the newborns was 17 days.

Conclusion

PA remains a serious materno-fetal emergency with a bad fetal prognosis for many newborns. Many fetuses either died or exhibited severe acidosis. Clinical signs and radiological images of PA are absent in many cases. There was more fetal acidosis for mothers who came from their homes at the time of delivery. We recommend that the delivery should not be delayed and a cesarean section must be the preferred mode of delivery. Pregnant women with vascular and metabolic diseases should be carefully monitored and informed on the risk of PA.

## Introduction

Placental abruption (PA) remains an emergency obstetrical situation that could jeopardize fetal and maternal lives [1, 2]. PA is often unpredictable and exhibits sudden onset [3].

PA occurs due to the premature detachment of a normally inserted placenta [3]. The basal decidual hematoma interrupts the maternal-fetal circulation and rapidly leads to hemodynamic disorders, coagulation abnormalities, and acute fetal distress [3]. It is a paroxysmal pathology of the last months of pregnancy and of labour.

PA leads to complications in about 0.4 to 1% of pregnancies [4, 5]. It is responsible for 5% to 10% of perinatal mortality and morbidity, and about 10% of cases with neurological disorders [6]. PA is also found in 5.1% of premature deliveries [2]. Some risks factors, such as maternal age (<20 or >35 years); large multiparity; thrombophilia; consumption of tobacco; alcohol; and cocaine; in vitro fertilisation (IVF); multiple pregnancies and vascular disorders; and premature birth or abnormalities of amniotic fluid, have been identified [3, 4, 6].

PA is responsible for metrorrhagia of black blood, abrupt and intense abdominal pain, uterine contracture, and sometimes fetal death. High blood pressure and proteinuria are usually exhibited by the mother; however, these symptoms are presented in only one-third of the cases [3]. Fetal heart rate abnormalities are present in 70% of PA cases [6].

The mothers could have a vascular collapse due to the hematoma. Coagulation troubles might arise along with a renal deficiency leading to a threat to the maternal life. Previous studies have also shown the use of ultrasonography to diagnose PA by diagnosing a retroplacental hematoma.

Retrospectively, the histopathological exam of the placenta can confirm the diagnosis but only in one-third of cases [4]. Indeed, specific lesions are not always present. Placental infarct and decidual vasculopathies are frequently observed in PA patients [7]. Many studies have shown the association between PA and poor fetal prognosis and maternal health. PA is the leading cause of cerebral palsy [8]. The diagnosis of PA remains difficult, and the fetal-maternal prognosis is inadequate in many cases. There are few recent studies on this topic. In our practice, we observed severe acidosis in newborns with placental abruption in some cases. 

With progress in maternal-fetal monitoring and neonatal resuscitation, we aimed to know the fetal prognosis in the case of PA and if the management of pregnant women diagnosed with placenta abruptio could be improved.

For this purpose, we investigated the means of diagnosis and management of PA in a tertiary maternity centre with neonatal resuscitation and fetal outcomes.

## Materials and methods

We conducted a retrospective study involving all PA cases admitted in the Maternity Department of the Regional Hospital Center of Orleans between January 1st, 2006 and December 31st, 2013. This centre performs approximately 4900 deliveries per year and has a neonatal resuscitation unit.

The data were anonymously extracted from medical charts. An anonymous number was attributed to each patient. We recorded general characteristics of patients, including age, parity, medical history, etc.

We also recorded obstetrics data related to PA, including metrorrhagia, pain, ultrasonographic exams of the placenta, fetal heart rate (FHR), birth weight, umbilical artery (UA) pH, and Apgar score. We also recorded the results of the macroscopic and histological examination of the placenta.

All pregnancies with >24 weeks of gestation period with PA suspected either clinically or diagnosed using ultrasonography and confirmed with macroscopic examination of the placenta by an obstetrician were included. If macroscopic exam of the placenta did not show hematoma and/or clots, the patients were excluded from the study.

Statistical analysis

Following a descriptive analysis (mean ± standard deviation or percentage + 95% confidence interval), we assumed that the sample size was big enough to use parametric tests for inferential statistics. Means were compared using one-factor analysis of variance (ANOVA). Where results were statistically significant, comparison by pairs was performed to calculate p-values and adjusted by Holm’s method. Relationships between continuous variables were analyzed to compute Pearson’s r correlation coefficient and 95% confidence interval. The software application used was R version 3.6.0 (R Foundation, Vienna, Austria). A p-value of 0.05 was considered statistically significant.

The study was approved by the Commission of Informatics and Liberty of the Hospital of Orleans (registration number: 2018-002).

## Results

During the study period, 201 patients out of 35814 who delivered were diagnosed with PA (0.56%). The mean age of patients was 30 years (17-46). Patients between 30 and 34 years of age were more frequently diagnosed with PA (34.8%). The majority of patients were of Caucasian origin (Table 1).

**Table 1:** General characteristics of patients suspected of placenta abruptio (n = 201) PRM: premature rupture of membranes; HIV: human immunodeficiency virus

		N	Percentages %
	Came from home	154	76,2
	Hospitalized	47	23
	Age (years)		
	< 20	5	2
	20-35	154	77
	35-40	34	17
	> 40	8	4
	Nulliparous	86	43
	Multiparous	115	57
	Pregnancy complications	151	75
	Vascular/ preeclampsia	34	17
	Gestational diabetes	4	2
	High Blood Pressure >140/90	102	51
	PRM	9	4
	Hydramnios	2	1
	Toxics and treatments	37	18
	Infections	5	2
	HIV	2	1
	C Hepatitis	2	1
	Chlamydia	1	0,5
	Neurological or psychiatric disorders	10	5
	Epilepsia	6	3
	Depression	3	1
	Anorexia	1	0,5

The mean parity was 2 (range: 0-8). The cases were mostly multiparous. No previous medical pathologies were observed for the majority of women (150 patients; 74.9%). Nine cases (4.5%) had a history of hypertensive disorder and 14 had previously had a C-section. Thirty-six patients (17.9%) smoked tobacco or drank alcohol during pregnancy. Majority of patients presented with pregnancy complications, mainly vascular and metabolic disorders, chronic infections, or neurological disorders (Table 2)

**Table 2:** Positive symptoms of placenta abruptio (n = 201) US exam PA: ultrasound examination of placenta abruptio

	n = 201	%
Metrorrhagia	117	58
Abdominal pain	79	39.1
Fetal heart rate analysis		
Abnormal	92	45.5
Plate rhythm	7	3.5
Intermediate	17	8.4
Normal	85	42.1
US exam PA	48	23.9
Positive histology (n = 144)	71	47.9

At the time of admission to the delivery room, 76.6% of cases came from their homes, while others were already hospitalized in the high-risk maternity unit for other reasons. The mean term of pregnancy at the time of diagnosis of PA was 35 weeks of gestation (WG) (range: 18-42 WG).

**Figure 1:**
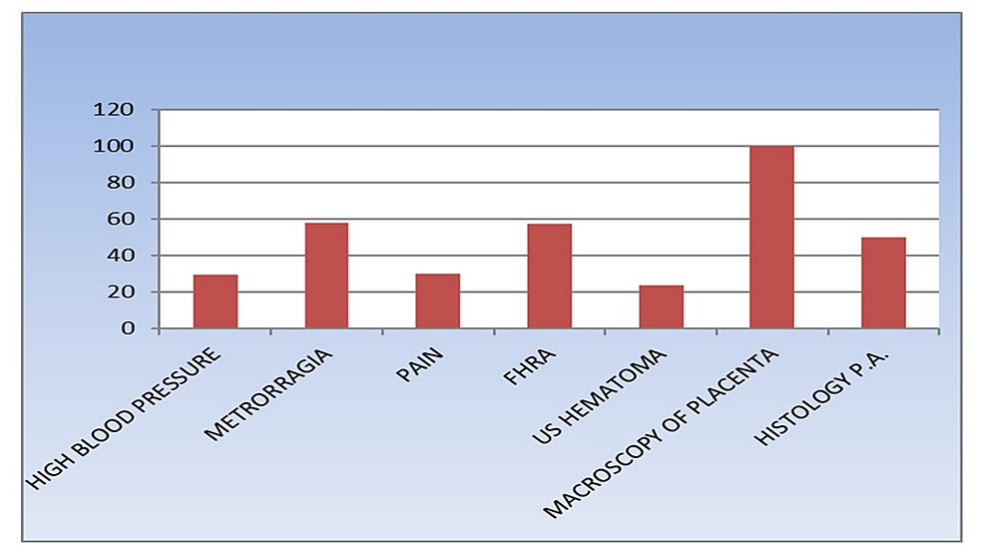
Diagnosis of placenta abruptio (n=201) FHRA: fetal heart rate abnormalities; PA: placental abruption; US: ultrasonography.

High blood pressure was exhibited by 60 patients (29.8%). Furthermore, 58% of patients presented with metrorrhagia and only 39% of patients complained of abdominal pain. Next, 114 fetuses (57%) were diagnosed with fetal heart rate abnormalities using cardiotocography (CTG) scale guidelines and were classified as pathological, intermediate, or plate (Figure 1).

One hundred and eighty patients (84.6%) underwent a C-section and 31 patients (15.4%) underwent a vaginal delivery. The mean time for delivery was 18.7 min.

Thirteen fetuses died at birth. Among them, five fetuses died in utero. Ninety-eight newborns needed neonatal resuscitation and were hospitalized at the neonatal intensive unit care.

One hundred sixty-seven fetuses were born prematurely. The mean birth term was 35.7 weeks. All patients, except 10, delivered under general or epidural anaesthesia. The mean birth weight was 2359 ± 861 g. The mean Apgar score was 6.23 at the first minute, 7.86 at the fifth minute, and 8.54 at the tenth minute.

Umbilical artery pH of newborns

In total, 31 fetuses presented with severe acidosis at birth with UA pH ≤7 (Table 3). Of them, 25 fetuses had UA pH <7 and six fetuses had UA pH = 7. Forty-five out of 188 alive newborns (24%) presented with metabolic acidosis or mixed acidosis (UA pH < 7.20)

**Table 3:** Umbilical artery PH according to delivery time

N	Mean Delivery time (min)	Standard deviation (min)	UA pH
25	17.88	4.31	<7
20	15.75	5.24	7-7.19
143	19.10	10.73	>7.2

Fourteen newborns had a UA pH between 7.1 and 7.2. The mean UA pH was 7.19 (range: 6.56-7.41). The mean UA pH was significantly lower for PA cases who came from their home compared to hospitalized women (p = 0.0015). There was a significant difference between delivery times of those who came from home and those who were hospitalised.

**Figure 2:**
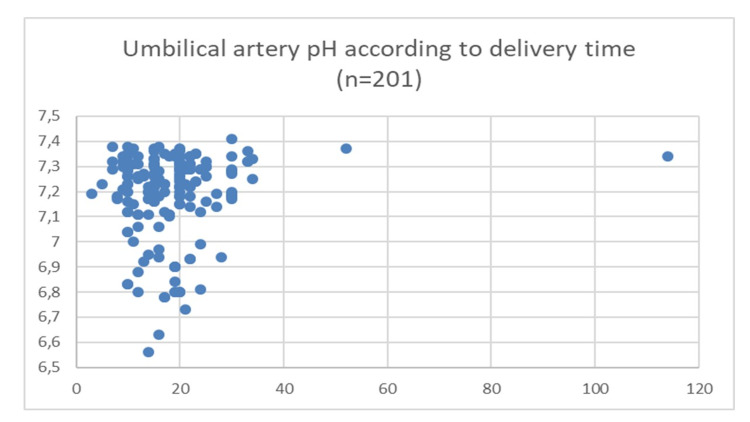
Umbilical artery pH according to delivery time

There was no significant difference in UA pH according to the time between the diagnosis of PA and the delivery (Figure 2). There was no Pearson correlation between the UA pH and the time to deliver, r = 0.12 (-0.02; 0.26) p = 0.09.

The mean stay in the neonatal intensive unit for the newborns was 17 days, with a mean gain weight of 625 grams. The sex ratio was 0.9:1 for females: males.

## Discussion

In our study, PA remains a severe pregnancy complication and sometimes a lethal complication. We reported many fetal deaths. One out of four fetuses presented with acidosis at the time of delivery. Although most deliveries occurred in a very short time, more than one out of ten fetuses presented a UA pH < 7. For Yamada et al. [8], in 104 cases of cerebral palsy, abruptio placenta was responsible for 26% of the cases. However, our neonatal mortality rate was inferior compared to some previous studies [9] and similar compared to others [10].

We did not observe maternal mortality among any of the cases. However, in Nigeria, for Igwegbe et al. [11], the rate of maternal mortality was 1/1000 in PA. Our situation is probably due to better means of anaesthesiology and resuscitation in France.

In our study, the mean UA pH was significantly lower for PA cases who came from their homes compared with hospitalised cases. The majority of patients came from their homes, which explains the bad fetal prognosis in many cases. Hospitalized patients were better prepared to have a C-section. In case of the presence of symptoms, obstetricians were in the hospital and they could decide very quickly to perform an emergency delivery. Therefore, the delay between the diagnosis and the fetal extraction is reduced in hospitalized patients compared to patients coming from their homes. For Yamada et al. [8], 22 of 28 mothers with PA delivered newborns with UA pH <7.

The emergency C-section was the main mode of delivery with a mean extraction time of 18 min similar to the reports of Matsaseng et al. and Matsuda et al. [12, 13] One out of three fetuses that were delivered through the vagina exhibited a UA pH < 7; therefore, vaginal delivery is not recommended except in case of imminent delivery. It increases the exposition to fetal hypoxia.

There was no significant difference in extraction time between fetuses with acidosis and those with normal UA pH. The absence of significant differences in UA pH of newborns according to the extraction time indicates that the PA is probably, in many cases, a subacute event with a progressive detachment of the placenta without any clinical manifestation during early stages. Thus, an emergency C-section will not completely resolve the problem, as, in many cases, fetal distress was present before the mother arrived at the hospital or before the apparition of clinical symptoms. In these situations, PA is probably misdiagnosed.

The characteristics of PA, its size, its location, and age of onset are the potential factors that elucidate the little impact of a quick extraction [7, 14]. These fetal extraction times, which are a little higher than those recommended in extreme emergencies, are linked to the difficulty of diagnosis of PA but also to the fact that most women came from their homes. Thus, the preparation time to have a surgery increased mildly. Also, 117 caesareans were performed in red code (emergency C section), with 24 newborns exhibiting UA pH ≤7.

Metrorrhagia, pain, and fetal heart rate abnormalities were found in only one-third to half of the patients suspected of PA. Hematoma during ultrasonography exam was only found for one out of four women. Pregnancy pathologies, like preeclampsia, vascular disorders, diabetes, coagulation troubles, cannabis or tobacco use and fetal growth restriction, were observed in many cases. For Shobeiri et al. [15], smoking and placenta abruption had a significant association.

Ultrasonography was used for all cases suspected of PA; however, only 24% of the cases were revealed to be suffering from PA, which corroborated the results of Tikkanen [3]. This finding might be attributed to the fact that, in the case of a recent hematoma, the diagnosis is difficult because the echogenicity of PA is not prominent and, at onset, PA could be small or little echogenic, thus difficult to visualize. Therefore, in case of suspicion of PA on the basis of clinical examination (preeclampsia, high blood pressure, diabetes, and coagulation troubles), fetal extraction should not be delayed.

Histological exam of the placenta confirmed the diagnosis of PA in about half of the patients. These results are in accordance with the fact that, sometimes, the anoxic lesions of the placenta, visualized as placental infarct, are not always present in acute forms. Other authors found the same results [4].

In our study, the prevalence of PA was inferior to that observed in African studies [16]; however, it was similar to that reported by other studies [5, 17]. The mean age of 30 years in PA cases was similar to other studies [11] along with the gestational age at the time of diagnosis [17].

Limitations of the study

Firstly, our study was retrospective. However, the data were well documented. In addition, it is very difficult to conduct such a study prospectively because of the very low incidence of PA. However, it is a large study on placental abruption and its management and fetal prognosis. Indeed, it included 201 patients. The results are important and showed means to improve the management of pregnant women with a suspicion of PA. Clinical and ultrasonographic images are absent in many cases.

Secondly, there was no case-control. However, the number of newborns with severe acidosis was very high and the number of newborns who died is very high compared to other deliveries without PA. Our study confirmed the bad fetal prognosis in many cases of PA even if the delivery time was short. Nevertheless, the fetal extraction time could be improved.

Indeed, in the case of suspicion of PA, Obstetricians should not waste too much time during the ultrasonographic exam. Patients with vascular and metabolic pathologies should be carefully monitored during their pregnancy.

## Conclusions

PA remains an extremely critical situation owing to high fetal morbidity and mortality. We did not observe any maternal mortality. Average fetal extraction time remained high in PA cases but could be improved. The clinical signs, metrorrhagia, pain, fetal heart rate abnormalities (FHRA), and placental hematoma at ultrasonography exam revealed inconsistent results. Most mothers with PA came from their homes and fetal acidosis was more frequent for mothers who came from home compared to hospitalised patients. Caesarean section should be the preferred way of delivery. Histological examination of the placenta revealed inconsistent results. Maternal vascular and metabolic diseases should be carefully monitored during pregnancy to prevent PA.
